# Mink Farms Predict Aleutian Disease Exposure in Wild American Mink

**DOI:** 10.1371/journal.pone.0021693

**Published:** 2011-07-18

**Authors:** Larissa A. Nituch, Jeff Bowman, Kaela B. Beauclerc, Albrecht I. Schulte-Hostedde

**Affiliations:** 1 Environmental and Life Sciences, Trent University, Peterborough, Ontario, Canada; 2 Ontario Ministry of Natural Resources, Trent University DNA Building, Peterborough, Ontario, Canada; 3 Biology Department, Laurentian University, Sudbury, Ontario, Canada; University of Kansas Medical Center, United States of America

## Abstract

**Background:**

Infectious diseases can often be of conservation importance for wildlife. Spillover, when infectious disease is transmitted from a reservoir population to sympatric wildlife, is a particular threat. American mink (*Neovison vison*) populations across Canada appear to be declining, but factors thus far explored have not fully explained this population trend. Recent research has shown, however, that domestic mink are escaping from mink farms and hybridizing with wild mink. Domestic mink may also be spreading Aleutian disease (AD), a highly pathogenic parvovirus prevalent in mink farms, to wild mink populations. AD could reduce fitness in wild mink by reducing both the productivity of adult females and survivorship of juveniles and adults.

**Methods:**

To assess the seroprevalence and geographic distribution of AD infection in free-ranging mink in relation to the presence of mink farms, we conducted both a large-scale serological survey, across the province of Ontario, and a smaller-scale survey, at the interface between a mink farm and wild mink.

**Conclusions/Significance:**

Antibodies to AD were detected in 29% of mink (60 of 208 mink sampled); however, seroprevalence was significantly higher in areas closer to mink farms than in areas farther from farms, at both large and small spatial scales. Our results indicate that mink farms act as sources of AD transmission to the wild. As such, it is likely that wild mink across North America may be experiencing increased exposure to AD, via disease transmission from mink farms, which may be affecting wild mink demographics across their range. In light of declining mink populations, high AD seroprevalence within some mink farms, and the large number of mink farms situated across North America, improved biosecurity measures on farms are warranted to prevent continued disease transmission at the interface between mink farms and wild mink populations.

## Introduction

Declines in carnivore populations are often associated with infectious diseases [1], [2]. Spillover, when infectious disease is transmitted from a reservoir population (often a domesticated species) to sympatric wildlife, is a particular threat to wild species because domestic animals can act as maintenance hosts [3]. Many cases of disease spillover from domestic animal reservoirs to wildlife have been reported, such as spillover of rabies from domestic dogs to the highly endangered Ethiopian Wolf (*Canis simensis*) [4], and repeated outbreaks of the Rinderpest virus in wild African ruminants caused by contact with domestic cattle [5].

American mink (*Neovison vison*) are an ecologically and economically important species, yet populations in Canada appear to have declined over the last 50 years [6]. Although many factors have been implicated as contributing to the declining mink population trend, including habitat loss [7], [8], overharvest, prey declines [9], and exposure to environmental contaminants, such as PCBs [10], these factors do not appear to fully explain mink declines [6], [11].

American mink have been domesticated since the late 1800s for the fur industry, and have likely been escaping into the wild since the advent of mink farming [12], [13]. Feral mink populations, resulting from deliberate releases by animal activists and accidental escapes from farms, have become widely established in Europe and South America (e.g., [14]–[16]). The negative effects on native biodiversity of feral mink, both through predation and competition, are well documented outside of North America (e.g., [17]–[20]).

Surprisingly, however, the ecological effects of domesticated American mink escaping from farms within their native range have been overlooked. With approximately 2 million mink housed in 221 fur farms across Canada [21], most of which are located in or near suitable habitat for wild mink [12] the potential for escaped domestic mink interacting with wild mink is high. Indeed, a recent analysis found that 64% of free-ranging mink in southern Ontario, Canada, were domestic or domestic-wild mink hybrids [22], clearly demonstrating that domestic mink are escaping from fur farms, surviving in the wild, and reproducing with wild mink. Escaped domestic mink may threaten native wild mink populations through hybridization, outbreeding depression, and competition for food, space, and mates [6]. Additionally, escaped domestic mink may pose a risk to wild mink populations through the transmission of new or elevated intensities of infectious disease.

Aleutian disease (AD), a highly pathogenic parvovirus affecting mink and other mustelids, is of particular concern. In adult mink, AD infection is characterized by hypergammaglobinemia, plasmacytosis, glomerulonephritis, decreased fertility, and spontaneous abortion and can lead to severe chronic immune dysfunction, increasing susceptibility to other diseases [23], [24]. In neonatal mink kits (typically those <10 weeks old), AD causes acute, rapidly progressing interstitial pneumonia with high mortality rates [25]. AD is transmitted horizontally by blood, feces, urine, and saliva or vertically from infected dams to their kits during the perinatal period [26]. Aleutian disease manifests in several forms; progressive infection, which is typically fatal; persistent nonprogressive infection, where mink remain healthy, but can still transmit virus causing progressive disease; and lastly, nonpersistent nonprogressive infection, where the virus is cleared from the host [27]. Disease intensity and progression varies depending on several factors, including host age, mink genotype, immune status of the host, and strain of the virus [23], [28].

AD is currently the most significant infectious disease affecting farmed mink worldwide, and the problem appears so severe in some regions that it may become a limiting factor in the industry's ability to produce mink [29]. Accurate data about disease prevalence in mink farms is scarce; however, a national voluntary survey of mink farms in Canada found that at the national level 32% (n = 15/47) of participating farms contained some AD seropositive mink [30]. Sample submission rates in this study were low, however, with 20% (47/238) of Canadian mink farms participating, and contributing an average of 200 samples per farm. Given that this sample was voluntary, estimates may be biased due to over- or under-reporting by AD-positive farms. Voluntary testing in the Canadian province of Ontario has found that the percentage of farms with AD positive reactors has been as low as 14% and as high as 60% between 1986 and 2006; although again, farm participation was low (Ontario Ministry of Agriculture, Food, and Rural Affairs, unpublished data).

The origin, prevalence and consequences of AD in free-ranging mink populations are even more uncertain. Feral American mink in Southern England [31], France [32] and Spain [33] have tested positive for AD antibodies. AD in Europe is suspected to have been introduced via imported domestic American mink from North America for the fur farming industry and may be contributing to population declines of native European mink [32]–[34]. In Canada, where American mink are native, the status of AD in the free-ranging population is unknown.

There are currently either no, or inadequate, regulations concerning the escape of farmed mink in Canada [6]. In most provincial jurisdictions, there are no minimum standards for biosecurity on fur farms. Perimeter fencing is often inadequate, and improper disposal of pelted mink carcasses, dead-stock, manure and other waste may be an important issue on many farms [29], [35]. AD virus is exceptionally hardy and can survive for 2 years or more in soil and improperly composted manure or carcasses [23]. As well, recent large-scale, intentional releases by animal rights activists in Ontario and Newfoundland have been from AD positive mink farms (Hunter, B., University of Guelph, pers. comm.).

Given the persistence of AD in mink farms, frequent farm escapes, a lack of biosecurity and waste management standards, and the hardy nature of the virus, the potential for mink farms to act as AD reservoirs and sources of AD transmission into the wild appears high. Transmission may be occurring both through the escape of domestic mink and through contact by wild mink with infected materials on mink farms. The introduction of AD to wild mink populations could reduce fitness in wild mink by reducing both productivity of adult females and survivorship of juveniles, leading to population declines.

To examine the potential role of mink farms and escaped domestic mink in the spread of AD to wild mink populations, we designed a two part study to assess the seroprevalence and geographic distribution of AD infection in free-ranging mink on a large-scale, across Ontario, and on a smaller-scale, at the interface between a mink farm and the wild. We hypothesized that domestic mink escaping from farms are a source of Aleutian disease virus (ADV) transmission to wild mink, which predicts that AD seroprevalence in the free-ranging population should be higher in feral mink than in wild mink. We also hypothesized that mink farms are a source of ADV transmission to wild mink, which predicts that AD seroprevalence in the wild should be higher in closer proximity to mink farms than in areas farther from farms. Lastly, considering the potential negative effects of AD on female productivity, we tested for variation in seroprevalence between sexes.

## Methods

### Ethics Statement

All mink were trapped and handled according to protocols approved by the Animal Care and Use Committees of Trent University and the Ontario Ministry of Natural Resources (OMNR), or, in the case of samples obtained through the fur harvest, in accordance with OMNR protocols and regulations.

### Large-scale survey

**Figure 1:**
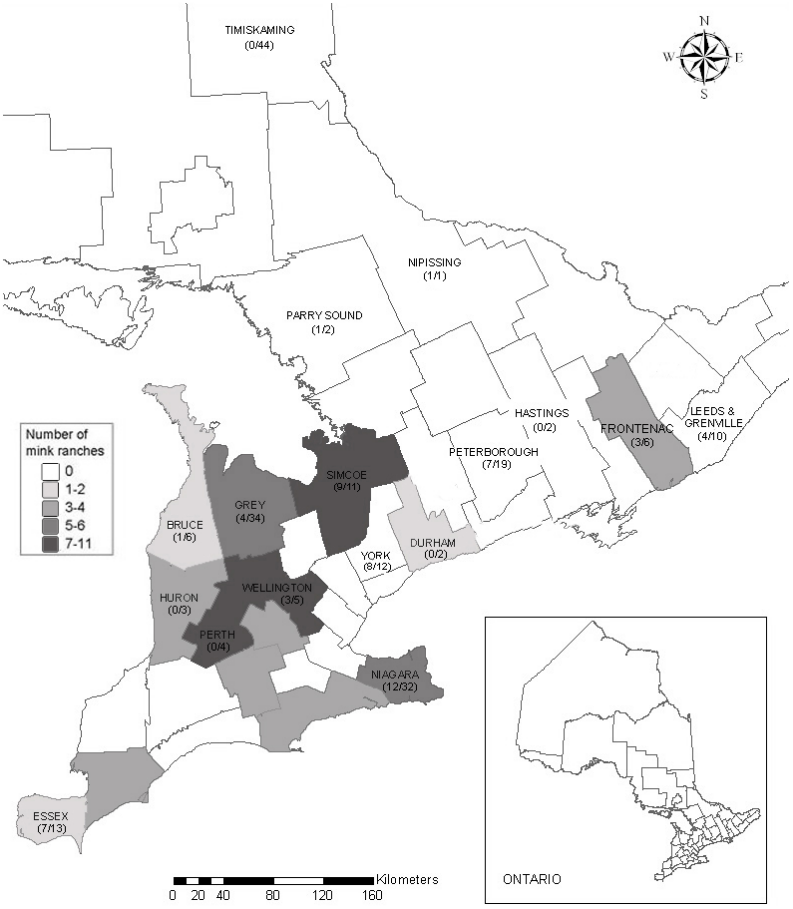
Counties in Ontario, Canada sampled for free-ranging American mink (*Neovison vison*) during winters 2005–2009 in a study of Aleutian disease seroprevalence. Mink farm abundance per county is noted by shading. Number of seropositive mink/number of mink sampled per county is noted in parentheses.

Free-ranging mink were collected, primarily by fur trappers, in 19 counties across Ontario during winters 2005–2009 (Fig. 1). Sampling was stratified according to mink farm density [21], with at least 3 replicates each of high, medium and low mink farm density counties being sampled. Trappers who provided carcasses reported the township where animals were caught; townships were rectangular political subdivisions embedded within counties and represented the finest resolution of sampling for our large-scale survey. During necropsy, we collected blood samples from each mink via cardiac puncture, using heparinized capillary tubes, which were then frozen at −20°C until tested. We also collected hair or muscle tissue for DNA analyses.

### Small-scale survey

Live trapping took place in a 596-km^2^ area of the Niagara peninsula, a high mink-farm density region, during July to November 2008. Mink were captured in Tomahawk live traps (Tomahawk Live Trap Co., Tomahawk, WI) baited with sardines. We targeted some trapping effort toward mink, but also included mink obtained from ongoing raccoon research in the study area. The combination of this targeted and untargeted trapping resulted in 65,720 trap nights of livetrapping effort. Data from the small-scale survey were also included in the large-scale analysis.

Captured mink were transferred, using trap dividers, to a holding cage where they were weighed and immobilized using a 10∶1 mixture of ketamine hydrochloride to xylazine hydrochloride at a dosage of 20 mg/kg of animal weight. All captured individuals were sexed, and standard body measurements were taken. Based on the timing of sampling, all mink should have been at least 4 months of age at the time of capture. We fitted each mink with a 1-g Monel ear tag, obtained a small hair sample for DNA analyses, and collected 2–5 mL of whole blood for antibody testing by clipping a toenail. Serum was then collected from the whole blood by centrifugation and immediately stored at −20°C until tested. After handling, xylazine was reversed with yohimbine at a dosage of 0.1 mg/kg, and mink were placed back into their traps until they were fully recovered from anaesthesia, at which point we released them at the site of capture.

Whole blood and sera samples from both surveys were analyzed for AD antibodies using the counterimmunoelectrophoresis (CIEP) test performed by the University of Guelph's Animal Health Lab (Guelph, Ontario). The CIEP test has a reported sensitivity of approximately 98%, specificity of 86–91% (9–14% false positives) and repeatability of 98–99% ([36]; Animal Health Lab, University of Guelph, unpublished data).

### Genotyping and data analysis

Mink were genotyped and assigned to population clusters (domestic, hybrid, and wild) according to methods described by Kidd et al. (2009). Individuals were assigned to populations with a minimum membership probability of q≥0.80, whereas all animals with q<0.80 for both groups were considered hybrids. Hereafter, we refer to these 3 genetic assignments as genotypes.

We used logistic regression to investigate the relationship between AD antibody status and mink genotype, sex, and distance from mink farms. Antibody status was numerically coded as 0 (CIEP negative) or 1 (positive). Mink genotypes were coded to 3 levels, where 0 = wild, 1 = hybrid, and 2 = domestic. Due to the deregulation of fur farming in Ontario, we could not determine precise locations of some mink farms. As such, for the analysis of large-scale seroprevalence, we estimated proximity of each mink to a mink farm by calculating the distance between the mink's township and the nearest township with a mink farm. For the smaller-scale analysis in Niagara, the precise mink farm location within the study area was known, therefore the distance from the site of capture to the mink farm was used.

Competing models for explaining patterns of AD exposure in our data were compared using Akaike's Information Criterion corrected for small sample bias (AICc) to assess evidence that distance from a mink farm, mink genotypes and sex influenced AD seroprevalence [37]. Our model set included each parameter singly, as well as all possible additive combinations. We considered models with delta AIC_c_ (difference between the AIC_c_ of each model and that of the top ranked model) of less than 2 to be our confidence set of models. For all variables in the confidence set, we calculated weighted model-averaged parameter estimates, standard errors, and Akaike importance weights [37]. We judged parameter estimates to be biologically meaningful if the 95% confidence interval based on the associated standard error did not contain zero. As well, for each variable in the confidence set, permutation tests were used to determine whether model effects were different from that expected by chance. Model variables were considered to have non-random effects if parameter estimates were greater than or less than 95% of permuted values (P<0.05 or P>0.95).

Chi-square analysis was used to test for differences in AD prevalence between sexes and mink genotype (domestic, hybrid or wild). Statistical analyses were carried out using either R [38] or Systat v.11 (Systat Inc. 2004, San Jose, CA, USA).

## Results

### Large-scale

Overall, CIEP-based seroprevalence of AD was 29% (*n* = 60/208) in free-ranging mink sampled across Ontario, with prevalence varying considerably between counties with a high density of mink farms and those with no mink farms (Fig. 1). For instance, in Simcoe, a county with high mink farm density, 9 of 11 (82%) mink were AD seropositive. Conversely, Timiskaming, which had no mink farms and was the farthest region sampled from any mink farms, had 0% seroprevalence (0 of 44 mink seropositive).

A total of 21% (44 of 208) of mink sampled were of domestic origin or domestic-wild hybrids. Hybrid mink had the highest seroprevalence (44% or 10/23), followed by free-ranging domestic mink (38% or 8/21) and wild mink with the lowest seroprevalence (28% or 42/164); however, this trend was not statistically significant (χ^2^ = 3.94, *p* = 0.142). Comparisons of overall seroprevalence between males (26%) and females (35%) were also not statistically significant (χ^2^ = 2.01, *p* = 0.157); however, captures were sex-biased with many fewer female mink (*n* = 75) being sampled than males (*n* = 133) (χ^2^ = 16.17, *p*<0.001).

Distance to the nearest mink farm was the best and most parsimonious model to explain large-scale AD seroprevalence in free-ranging mink (Tables 1 & 2). The next most important model was distance + mink genotype We averaged the confidence set of candidate models and found that distance (β_distance_ = −0.011; 95% CI: −0.004 to −0.017; Importance = 1.00) was 2.9 times more important than the next most important variable, mink genotype (β_population_ = 0.288; 95% CI: −0.382 to 0.958; Importance = 0.342). Permutation tests demonstrated that all variables in the confidence set had coefficients that were significantly different from random (Table 2). Confidence intervals for the slope of the mink genotype variable spanned zero however, suggesting only weak support for the effects of mink genotype on AD seroprevalence. The effects of sex on AD seroprevalence appear to be negligible as the parameter had both low importance value and confidence intervals that spanned zero. McFadden's rho^2^ of the global model was 0.13 (χ^2^ = 11.61, *n* = 208, *p* = 0.022).

**Table 1:** Candidate models for logistic regression analyses explaining large- and small- scale Aleutian disease seroprevalence in free-ranging mink across Ontario.

Large-Scale	Model Statement	AIC_c_	ΔAIC_c_	w_i_
	Distance^a^	222.68	0.00	0.569
	Distance + Genotype^b^	223.99	1.31	0.296
	Distance + Sex	226.40	3.72	0.088
	Distance + Genotype + Sex	227.68	5.00	0.047
	Genotype	252.66	29.98	0.000
	Sex	252.95	30.27	0.000
	Intercept	253.33	30.65	0.000
	Genotype + Sex	253.44	30.76	0.000

Small-Scale	Model Statement	AIC_c_	ΔAIC_c_	w_i_
	Distance^c^	37.43	0.00	0.581
	Distance + Genotype	39.65	2.22	0.191
	Distance + Sex	40.07	2.64	0.156
	Distance + Genotype + Sex	42.13	4.70	0.055
	Genotype	46.29	8.86	0.007
	Sex	46.26	8.83	0.007
	Intercept	46.47	9.04	0.003
	Genotype + Sex	48.17	10.74	0.003

a Distance = Distance from the centroid of the township of capture to the centroid of the nearest township with at least one active mink farm.

b Mink genotype (domestic, hybrid or wild).

c Distance = Distance from capture site to the mink farm.

For each model, Akaike's Information Criterion corrected for small sample sizes (AIC_c_), the difference between AIC_c_ of the top model and model *i* (ΔAIC_c_), and Akaike weights (w_i_) are shown.

**Table 2:** Parameter estimates, standard errors, 95% confidence intervals, and permuted P-values (1,000 iterations) for the confidence set (ΔAIC_c_ <2) of models explaining large- and small- scale Aleutian disease seroprevalence in free-ranging mink across Ontario.

Large-Scale	Parameter	Estimate	SE	LCL	UCL	p
*Distance Model*	Distance^a^	−0.013	0.003	−0.019	−0.007	0.005
	Intercept	−0.279	0.185	−0.642	0.084	0.009
*Distance + Genotype Model*	Distance	−0.021	0.004	−0.029	−0.013	0.005
	Genotype^b^	0.306	0.332	−0.345	0.957	0.001
	Intercept	−0.317	0.174	−0.658	0.024	0.015

Small-Scale	Parameter	Estimate	SE	LCL	UCL	p
*Distance Model*	Distance^c^	−0.134	0.058	−0.248	−0.020	0.001
	Intercept	−0.331	0.264	−0.848	0.186	0.000

a Distance = Distance from the centroid of the township of capture to the centroid of the nearest township with at least one active mink farm.

b Mink genotype (domestic, hybrid or wild).

c Distance = Distance from capture site to the mink farm.

### Small-scale

In the Niagara peninsula, 38% of mink (*n* = 13/34) were AD seropositive. Sixty-two percent of the free-ranging population consisted of domestic or domestic-wild hybrids, highlighting a potential biosecurity issue on farms in this region. On this smaller scale, once more, we noted that seroprevalence was highest in hybrid mink (67%; 4 of 6 mink seropositive), moderate in escaped farm mink (40%; 6 of 15) and lowest in wild mink (23%; 3 of 13); however, again this trend was not significant (χ^2^ = 2.34, *p* = 0.306).

The model including distance to farm was most strongly supported by the data, and was the only model in the confidence set (Tables 1 & 2). Seronegative mink were caught, on average, 10 km (95% CI, 13.1 – 7.4 km) from the mink farm. Seropositive individuals were caught an average of 4.5 km (CI, 6.5 – 2.5 km) from the mink farm. The permutation test for distance was significant, suggesting that its parameter estimate was different than that expected by chance (Table 2). All other variables had low importance values, and confidence intervals that crossed zero, indicating that we did not detect effects of mink genotype or sex on AD seroprevalence at the small spatial scale. McFadden's rho^2^ of the global model was 0.17 (χ^2^ = 32.78, *n* = 34, *p*<0.001).

## Discussion

The identification of significant reservoirs of disease is fundamental to the management of disease transmitted between wildlife and domestic livestock [39]. We found that AD is present and widespread among free-ranging mink in Ontario. Our first hypothesis, that AD is spread by mink escaping from farms, was only weakly supported, and only at the large scale. We found very little evidence of a relationship between AD seropositivity and mink genotype (domestic, hybrid or wild). Instead, our findings were more consistent with our second hypothesis, that mink farms themselves are sources of AD virus. Seroprevalence in free-ranging mink was higher in closer proximity to mink farms at both scales of investigation. Distance from the nearest mink farm was a stronger predictor of AD seroprevalence at the small-scale interface between the farm and the wild (Niagara) than at the larger scale of investigation (Ontario). We interpret our findings to suggest that AD was spreading from point sources (mink farms) into the free-ranging mink population. At the small spatial scale, the signature of this pattern was relatively strong, when we sampled close to the sole active mink farm present within the study area. At the larger spatial scale however, AD prevalence appeared to take on a less structured spatial pattern of seroprevalence, which was linked to multiple point sources (multiple farms). Although most of our samples from northern Ontario, where there are currently no mink farms, were obtained from one single county (Timiskaming), we do not believe this has skewed the effect of distance on seroprevalance, as the Timiskaming samples actually originated from 7 different townships. Moreover, we observed the same trend of higher seroprevalence in closer proximity to mink farms at the smaller scale of investigation, in which Timiskaming samples were not included. A similar pattern has been noted resulting from spillover of the pathogen *Crithidia bombi* from commercially reared bumble bees to wild bumble bees (*Bombus* spp.), where the prevalence and intensity of infections in the wild declined with increasing distance from commercial greenhouse operations [40].

On farms, AD could be transmitted to the free-ranging mink population through direct contact between wild and farmed animals, contact by wild individuals with contaminated carcasses and waste, or through aerosol dispersal. Commercial farms are potential sources for the maintenance and spread of infectious disease because animals are often kept at continuously high population densities (for example, some mink farms house >10,000 mink), and new animals are regularly imported from other sources, increasing the potential for infectious diseases to flourish [41], [42].

Seropositive mink, escaped domestic mink and wild-domestic hybrids were found in most counties sampled, including those without any known active mink farms, highlighting the extensive geographic extent of the impact of mink farming. Additionally, we observed with radio telemetry (L.A. Nituch and J. Bowman, unpublished data) several escaped domestic mink, including some that were AD seropositive, making long range movements (as far as 29 km in <2 weeks), which could potentially enhance their role as vectors for the spread of the virus.

The historical occurrence of AD in free-ranging mink in Ontario and the rest of North America remains in question. Ours is the first large-scale field study of AD in free-ranging mink within their native range. It is unknown whether AD circulates within wild mink populations; the only previous documented cases of AD in wild mink in North America were recorded during 1978 in northern Ontario, in an area ≤48 km from several mink farms [43]. The disease was first identified in captive mink in the 1940s [44], and mink have likely been escaping from farms across North America since the beginning of fur farming. As such, it is highly likely that this problem has been long-term, and that wild mink across North America may be experiencing increased exposure to AD, via disease transmission from mink farms, which may have already had demographic consequences for wild mink across their range.

In order to prevent continued disease transmission between mink farms and wild mink populations, there are several steps that could be taken to improve biosecurity. For example, mink farms could require licenses that are conditional on a set of minimum standards, such as the use of proper climb-proof fences around mink farms, safe disposal of pelted carcasses, and measures to prevent potentially ADV contaminated waste from seeping into nearby groundwater. In Denmark, where feral mink are invasive, legislation ensures that all mink farms are surrounded by fences with a minimum height, buried bottoms, and smooth boards [12]. As well, proper screening and quarantine of new animals, and improved farm cleaning and disinfection methods, are critical to prevent disease [29]. Enhanced biosecurity on farms would not only benefit wild populations adjacent to farms by preventing further spillover of AD, but would also be advantageous to farmers, who can suffer large financial costs due to AD outbreaks, by preventing “spill-back” infections from wild and feral mink back to farms.

Repeated introduction of AD to wild populations from escaped domestic mink and mink farms is of serious concern as it may be contributing to the long-term and sustained decline of native mink populations through direct mortality of adults, as well as by reducing both productivity of adult females and survivorship of juveniles. Once introduced into the wild, AD cannot be eradicated by traditional control methods as there is presently no effective treatment or vaccine [45]. Thus, controlling the disease in maintenance hosts and preventing further transmission to native populations should be a priority for conservationists and policy-makers alike.
